# New Nanosized V(III), Fe(III), and Ni(II) Complexes Comprising Schiff Base and 2-Amino-4-Methyl Pyrimidine: Synthesis, Properties, and Biological Activity

**DOI:** 10.1155/2024/9198129

**Published:** 2024-05-14

**Authors:** Maged S. Al-Fakeh, Maha A. Alsikhan, Jawza S. H. Alnawmasi, Abdullah H. Alluhayb, Mona S. Al-Wahibi

**Affiliations:** ^1^Department of Chemistry, College of Science, Qassim University, Buraydah 51452, Saudi Arabia; ^2^Taiz University, Taiz 3086, Yemen; ^3^Department of Botany and Microbiology, College of Science, King Saud University, Riyadh 2455, Saudi Arabia

## Abstract

A new synthesis of mixed ligand complexes vanadium(III), iron(III), and nickel(II), [M : L1 : L2], where L1 = Schiff base 2-((E)-((4-(((E)-benzylidene)amino)phenyl)imino)methyl)-naphthalene-1-ol (C_24_H_18_N_2_O) as for L2 = AMPY 2-amino-4-methyl pyrimidine (C_5_H_7_N_3_) were prepared in powder and investigated. Element analysis, molar conductivity, FT-IR, UV-vis, and magnetic susceptibility values have been acquired to describe the generated complexes. The values of vanadium(III), iron(III), and nickel(II) compounds are, respectively, 2.88 BM, 5.96 BM, and 2.92 BM, demonstrating that all compounds conform to the recommended octahedral geometry. Thermal gravimetric analysis (TGA) is used to further assess the complexes and establish the temperature stability and degradation of the metal complexes. The calculations abstracted from XRD patterns propose nanosized complexes (average size 29–50 nm). The microstructures of the samples have also been investigated by scanning electron microscopy (SEM). The disc diffusion method was used to assess and analyze the inhibition of the growth of compounds against harmful bacterial and fungal strains. The prepared complexes were tested against three strains of bacteria, one gram-positive strain (*Bacillus subtilis*), two gram-negative strains (*Escherichia coli* and *Pseudomonas aeruginosa*), and one fungus (*Aspergillus fumigatus*). The complexes inferred antimicrobial activity against the studied organisms. Specifically, vanadium(III) and nickel(II) are more effective than iron(III), making them promising drugs.

## 1. Introduction

**Figure 1:**
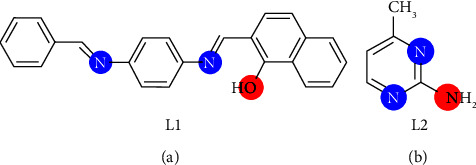
Chemical structure of (a) Schiff base 2-((E)-((4-(((E)-benzylidene)amino) phenyl)imino)methyl)-naphthalene-1-ol. (b) 2-amino-4-methyl pyrimidine.

The coordination complexes are made up of a core, an ion or atom, which is typically metallic, and an array of bonded ions or ions that are known as ligands or complexing agents. Transition metals are frequently found in coordination complexes [1]. A wide range of structural architecture and high design abilities are supplemented (available here) by using transition elements and organic ligands in chemistry as their building components [2]. This group of complexes has a wide range of applications in analysis, homogeneous catalysis, bioinorganic chemistry, and other fields. Because organic heterocyclic ring complexes have numerous uses in agriculture, health, pharmacy, and other industries, heterocyclic complex chemistry is a growing area of biology and chemistry [1]. Mixed ligand complexes are different from classical complexes in that they pair the same metal ion with at least two different kinds of ligands. This increases the potential for shifting properties, increasing the researchers' interest in synthesizing mixed ligand complexes. Hence, mixed-ligand complex synthesis and characterization have drawn a great deal of attention in recent years. It is common knowledge in the scientific community that mixed ligand complexes are crucial to the functioning of biological systems. It has been demonstrated that these complexes are biologically effective against a range of harmful microorganisms. In coordination chemistry, organic bidentate ligands are a frequent form of ligand and have a variety of applications. Oxygen and nitrogen are examples of donor atoms found in ligands. Complexes with diverse geometrical features are physiologically active as a result of their interaction with metal ions [3]. Interest in the structural chemistry, catalysis, and biological properties of transition-metal complexes containing heterocyclic molecules has been high. Amazing progress has been made in the field thanks to the synthesis of multidentate ligands from heterocyclic compounds containing metal ions. In coordination chemistry, a variety of multidentate ligands have crucial functions to perform. Numerous attempts have been made to create multidentate ligands. Schiff bases include a number of these ligands that have a C=N (azomethine) group. They were given the name Schiff bases in honor of their discoverer, German Hugo Schiff, who discovered in 1864. Simple condensing of primary amines and carbonyl compounds was used to make them [4]. Here, azomethine nitrogen and phenol oxygen act as coordination partners and serve as coordination partners; it has been shown in tests that Schiff's base is a superior ligand because it possesses a flexible, toothed structure [5]. The C=N connection plays a significant role in the demonstration of biological activity, due to the discovery that the lone pair of electrons of the nitrogen atom of the azomethine group in a sp^2^ hybridized orbital has chemical and biological significance [6]. In the chemistry of metals, coordination Schiff bases are considered “privileged ligands,” which means that they are employed in a variety of industries and fields, including chemistry, organic synthesis, analytical, and optical and electrochemical sensors. In addition, transition metal complexes of Schiff bases are also used in the food, leather, and wood dyeing industries. It also represents a wide range of medicinal chemistry applications. A wide range of biological activities, including antifungal, antibacterial, antitumor, tuberculosis, antidiabetic, analgesic, antispasmodic, antiproliferative, antimalarial, anti-inflammatory, antiviral, and antipyretic characteristics, have been demonstrated in them due to the wide range of pharmacokinetic characteristics and popularity in medication development [7–9]. The ability of Schiff bases to automatically and spontaneously form a monolayer, with a layer on the surface to be protected, makes this one of their most significant characteristics [10, 11]. Furthermore, pyridine derivatives serve as useful chelating ligands, and the complex-forming ability of pyridine and its derivatives with transition metal ions is well known, where they function as monodentate ligands [12]. Furthermore, the amino group (NH_2_) has been reported in several papers related to pyridine derivative compounds [13]. Pyrimidines are a heterocyclic ring with remarkable biological activity, which has been studied extensively because of its occurrence in living systems. Many pyrimidines and their derivatives have been used in various fields ranging from medical applications to industrial applications. Knowledge of the coordinating characteristics of the pyrimidine ring system is crucial for understanding the function of metal ions in biological systems since it offers a potential binding site for metals [12]. The chemotherapy drug bleomycin is a useful example of pyrimidine activity. Antimicrobial, antimalarial, antispasmodic, antineoplastic, and antidiabetic and nucleic acids (cytosine and uracil) [13]. These organic compounds (pyrimidines and their derivatives) are crucial since a repeated take of these drugs can lead to significant neurotic changes that affect the immunological function of the body and increase the generation of antibodies [14]. The dimethyl pyrimidine derivative-based HIV-1 CCR5 entry inhibitor (vicriviroc), nifekalant, is a category III antiarrhythmic drug, and urapidil is an antihypertensive sympatholytic drug [13]. Large proportions of people die from microbial infections and among the common microbial species that threaten human civilization and pose a danger, such as *Bacillus subtilis*, *Escherichia coli*, *Pseudomonas aeruginosa*, and *Aspergillus fumigatus*, which were used in this study. By measuring the diameter of the inhibition zone (IZ) surrounding the hole (mm), antimicrobial activity was assessed. According to the inhibitory zones discovered and the literature, all complexes are efficient against the four species [15–17] in that all of them showed better activity for the metal complexes compared to the original ligands (Schiff base and AMPY) as expected. The antimicrobial activities of the studied complexes were found in the following order: metal complexes > AMPY ligand > HL ligand. The findings demonstrate that coordination with the metal atoms increases the activity of the Schiff base and it explains that, when complexation is performed, the polarity of the metal atom will be reduced by partial sharing of its positive charge with donor groups and possible *π*-electron delocalization throughout the ring. This increases the lipophilic character of the complex and favors the permeation of the complex through the lipid layer of the cell membrane. The complex blocks metal binding sites in microorganism enzymes. As a result, they have the ability of metal complexes to inhibit microbial growth more effectively than free ligands [18, 19]. Previously, compounds of chromium(III), manganese (II), copper(II), and zinc(II) were reported to show antibacterial activity, anticancer, and antioxidant [3, 4]. Therefore, we speculate that compounds of V(III), Fe(III), and Ni(II) with 2-amino-4-methyl pyrimidine and Schiff's base will show more antibacterial, anticancer, and antioxidant activity. The preparation and characterization of mixed ligand complexes of vanadium(III), iron(III), and nickel(II), with a 1 : 1 : 1 (M : L1 : L2) ratio, are the subjects of the study given in this publication. The Schiff base, aminopyrimidyl, and its complexes were studied using elemental analysis, IR, ultraviolet-visible analysis, conductivity measurement, magnetic behavior, XRD, and thermal analysis (Figures 1(a) and 1(b)) and show the ligands employed in this investigation, HL and AMPY, respectively.

## 2. Experimental

### 2.1. Materials and Methods

This work's chemicals are 2-hydroxy-1-naphthaldehyde, p-phenylenediamine, and benzaldehyde, 2-amino-4-methylpyrimidine with excellent purity 97%, ethanol 99.7–100%, methanol, and dimethyl sulfoxide (DMSO). They were purchased and used without purification. The commercially available salts VCl_3_, FeCl_3_.6H_2_O, and NiCl_2_.6H_2_O were used without additional purification. Concentrations of carbon, hydrogen, and nitrogen were measured using a Euro vector CHN analyzer (EA3000, Italy). Agilent Technologies (Cary 600 Series FT-IR Spectrometer) was used to measure the solid-state FTIR spectra of the ligand and its complexes spanning the wave range 400–4000 cm^−1^. A magnetic susceptibility balance (MSB-Auto) from Sherwood Scientific was used to detect magnetic moments (the United Kingdom). The absorption spectra of the solutions (DMSO, 1 × 10^–3^ M) were also captured using a Shimadzu 1650-Spec UV-vis spectrometer (Shimadzu, Duisburg, Germany). At temperatures ranging from 20 to 600°C, the compounds were thermally examined in dynamic air using a Shimadzu (DTG 60-H) thermal analyzer. The control unit for the Philips XRD model (PW 1710) uses a Cu K anode at 40 K. V 30 M. (*l* = 1.54180). SEM (JEOL JSM-5400-LV field Emission SEM) was used to conduct the research, and the morphology of the complexes was investigated through it.

### 2.2. Antimicrobial Activity Test

#### 2.2.1. Antibacterial Assignment

The in vitro antibacterial sensitivity of each of the complexes was tested using the disc diffusion method [20]. Nutrient agar medium (28 g) in 1000 ml of distilled H_2_O was then poured into Petri discs and allowed to solidify for 15 min in a sterilized zone, and plates were incubated overnight with the following bacterial strains: Bacillus Subtilis (+ve) and Escherichia coli, Pseudomonas aeruginosa (–ve). These were obtained from the Department of Botany and Microbiology, King Saud University. The NPs of vanadium(III), iron(III), and nickel(II) used in the study were aliquoted until the wells were filled. The resulting mixture was stirred until it reached incubation for 24 hours at 37°C. Then, the diameter of the damping zone was measured.

#### 2.2.2. Antifungal Assignment

Antifungal activity studied: *Aspergillus fumigatus*. This sample was obtained from the Department of Botany and Microbiology, King Saud University. The PDA medium was then prepared and sterilized. 2 ml of treatment was added to 20 ml of PDA medium in a Petri dish, and the mixture was homogenized. This was followed by the transfer of a fungus disc, using an inoculation needle, and placing it upside down in the middle of the plate. The samples were then placed in a party dish, which was incubated for 5 days at 27°C. After that, the results were captured with a camera. The diameter of the fungal growth was measured from the plate directly using a ruler, and the results were recorded and compared with the control experiment.

### 2.3. Synthesis of the Schiff Base Ligand (HL)

**Figure 2:**
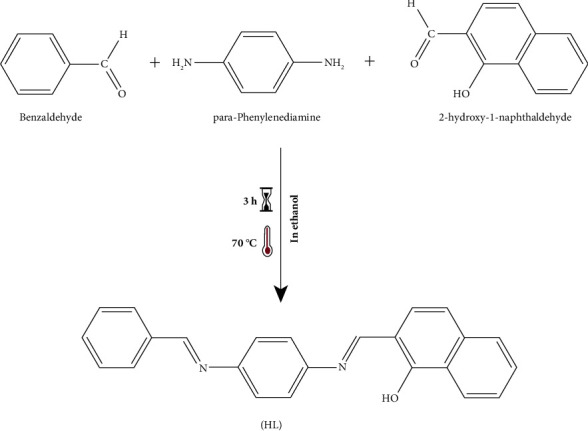
Synthesis of the (HL) ligand.

The ligand was made using the process described in the literature [4], in which 2-hydroxy-1-naphthaldehyde (3.5 g) and benzaldehyde (3.1 g) his solution were added dropwise to a flask containing (2.5 g) of p-phenylenediamine dissolved in ethanol. During the three hours that a mixture was allowed to stir while experiencing reflux to form an orange precipitate, the precipitate was filtered, repeatedly, rinsed with distilled water, and then completely EtOH (Figure 2).

### 2.4. Preparation of Metal Complexes

#### 2.4.1. [V(HL)(AMPY)(H_2_O)Cl_2_] (1)

**Figure 3:**
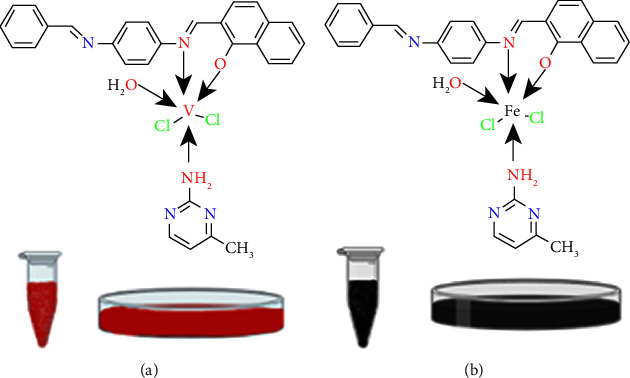
Structures of (a) V(III) and (b) Fe(III) complexes.

The V(III) complex was synthesized by 0.134 g (0.8 mmol) of (VCl_3_) was dissolved in 15 ml of distilled H_2_O and added to an ethanolic solution (20 ml) containing 0.3 g (0.8 mmol) of 2-((E)-((4-(((E)-benzylidene)amino)phenyl)imino)methyl)-naphthalene-1-ol (HL) ligand. Next, the addition of the second ligand 2-amino-4-methyl pyrimidine (AMPY) 0.093 g (0.8 mmol) in 10 ml of methanol. The mixture was stirred for 3 h at 60°C. After the refluxing procedure, the mixture was allowed to cool to room temperature. A red product precipitated, which was filtered and cleaned with distilled water and ethanol before being placed in the oven to dry for two hours at 50°C (Figure 3(a)).

#### 2.4.2. [Fe(HL) (AMPY) (H_2_O)Cl_2_] (2)

The same approach and method as in (1) and the same number of ligands were used. FeCl_3_.6H_2_O (0.231 g) 0.8 mmol. The final product was black red powder.‏ ‏Figure 3(b) shows the structure.

#### 2.4.3. [Ni(HL) (AMPY) (H2O)Cl2] (3)

**Figure 4:**
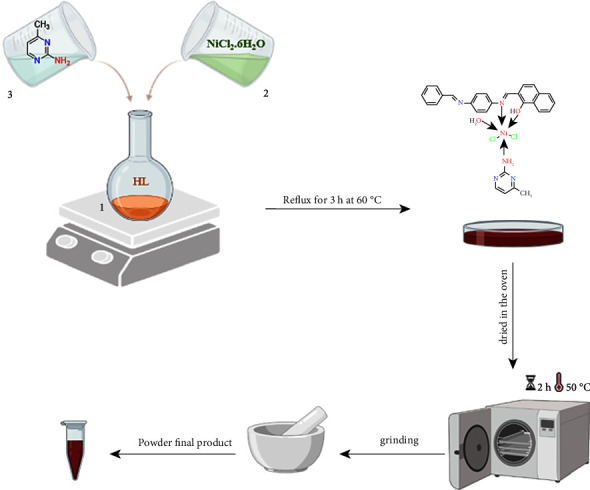
Preparation of the Ni(II) complex.

The same steps are there as in (1) and (2). NiCl_2_.6H_2_O (0.317 g, 1.3 mmol), HL (0.468 g, 1.3 mmol), and AMPY were all combined in a 1 : 1 : 1 molar ratio (0.145 g, 1.3 mmol) produced brick red powder in the end (Figure 4).

## 3. Results and Discussion

### 3.1. Elemental Analysis and Physical Properties of the Ligand and Complexes

In Table 1, the information from the ligand and its complexes' element analysis (CHN) is displayed in a 1 : 1 : 1 [M : L1 : L2] ratio. The values obtained and those calculated using the suggested formula agree with the formula of the vanadium, iron, and nickel complexes. Table 1 displays certain physical characteristics (color and melting points) for HL, AMPY, and their complexes. These characteristics demonstrate that the complexes have different colors from those of the ligands and have a high melting point, which is greater than the melting point of the ligands. This demonstrates that the coordination between the ligands and their metal salts, leading to complexation, is present. In DMSO solvent, the molar conductivity of the mixed ligand chelates was measured, and the results are given in Table 1. Low values for complexes of metal ions point to nonelectrolytic activity.

### 3.2. Fourier Transform Infrared Spectra

**Figure 5:**
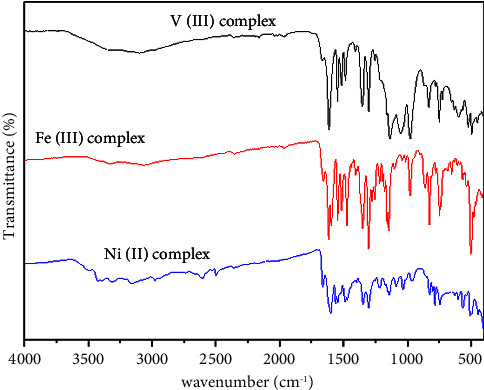
IR spectrum of V(III), Fe(III), and Ni(II) compounds.

The detailed assignment of all bands is based on the comparison with the significant frequencies in the IR spectra of the free ligands HL and AMPY with metal complexes (Table 2). The intraligand hydrogen bonding that the phenolic (OH) bond engages in with an azomethine nitrogen atom's (OH…N) lone electron pair prevents the ligand 1 (HL) FT-IR spectra from revealing the stretching frequency of the phenolic (OH) bond. The two azomethine groups (C=N) have strong bands at 1618,1604 cm^−1^ in all complexes, while the azomethine group (C=N) experiences a change that requires coordination with the metal center. In the phenolic (CO) bond, undergoing stretching at a frequency of 1310 cm^−1^, the phenolic OH group is involved in chelation because the stretching frequency of the phenolic (CO) bond has changed in the stretching band of the spectra of the produced complexes. The development of new peaks in the complexes as a result of (OH) rocking lends additional credence to this chelation. The (C=C) medium band appears at 1540 cm^−1^ [4]. Ligand 2 (AMPY) coordination occurs in the nitrogen amino. Stretching and bending modes for NH_2_ change in free ligand 3430−3305 cm^−1^ to lower wave numbers as in the Ni(II) complex when the nitrogen amino is engaged in complex building, which also disappeared in C1 and C2, sometimes the complex spectra contain water coordination, which makes it challenging to interpret them since the NH_2_ group of the AMPY ligand will overlap with that of the water molecules. The *δ*(NH_2_) vibration of NH_2_ provides more proof that the NH_2_ group is involved. The change in the wavelength of this band between the complexes and the free ADMPY ligand from 1648 cm^−1^ to 1656–1665 cm^−1^ indicates that the NH_2_ group was involved in the complex formation [4, 21]. At a wavelength of 3520–3030 cm^−1^, the coordinated water is indicated by the stretching vibration of coordinated H_2_O (OH) in the complexes. The bands that emerge at 612–640, 544–566, and 419–442 cm^−1^, respectively, are indications of the M-O, M-N, and M-Cl bonding, respectively (Figure 5) [4].

### 3.3. Electronic Spectra Electronic Spectra and Magnetic Moments

**Figure 6:**
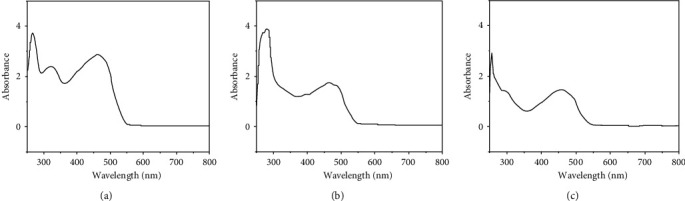
UV-vis spectrum of (a) V(III), (b) Fe(III), and (c) Ni(II) compounds.

The arrangement of the ligand in metal complexes is possibly inferred to from the electronic spectra (Figure 6). Additionally, it makes a distinction between different geometries. The magnetic moment value provides accurate details on the complex's shape and paramagnetic or diamagnetic nature. The ligands (HL) and (AMPY) showed two bands at 38,610 cm^−1^ returning to the transformation *π* ⟶ *π*^*∗*^ and 35,714 cm^−1^ attributed to the transition *n* ⟶ *π*^*∗*^ [4, 22, 23]. The strongest indication that coordination took place was the appearance of displacement. The magnetic moment values and UV-visible spectral data are provided in Table 3. Vanadium (III) exhibits a band at 20,492 cm^−1^, which may be assigned to ^3^T_1_ (F) ⟶ ^3^T_1_ (P) transition in the octahedral geometry of the complex [24], magnetic moment for V(III) (2.88 BM) [25]. Three absorption bands of the iron(III) complex can be seen at 20,450, 21,598, and 25,641 cm^−1^, and these are values related to transfers ^4^T_1g_ (D) ⟶ ^6^A_1g_, ^4^T_2g_ (G) ⟶ ^6^A_1g_, and ^4^T_2g_ (G) ⟶ ^6^A_1g_, serially [26, 27]. The magnetic moment shows the iron (III) (5.96 BM) [28], which indicates the presence of the Fe(III) complex in the octahedral structure. The electronic spectra of the nickel(II) complex exhibited one band of the d-d transition at *λ*max 21,277 cm^−1^, and these correspond to the ^3^A_2g_ (F) ⟶ ^3^T_1g_ (P) transition [29]. The Ni(II) complex was shown to possess two unpaired electrons and is paramagnetic because its octahedral geometry agrees with its magnetic moment value of (2.92 BM) [29].

### 3.4. Thermal Analysis

Thermal analyses of the manufactured complexes were performed up to 550°C. Using this method, complex composition, temperature stability, and the presence or absence of water molecules inside or outside the compound's inner coordination sphere can all be determined. TGA thermograms of the synthesized ligand and transition metal polymeric complexes showed a gradual weight reduction, indicating disintegration by fragmentation as the temperature increased. The findings revealed a good agreement between the calculated data and the suggested formulae for weight loss (Table 4).

#### 3.4.1. [V(HL)(AMPY)(H_2_O)Cl_2_]

**Figure 7:**
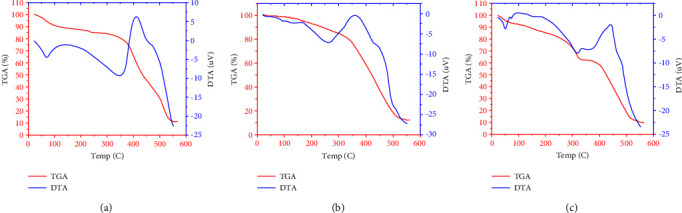
TGA and DTA curves of (a) V(III), (b) Fe(III), and (c) Ni(II) compounds.

The thermogram indicates that there are *four* distinct steps of mass loss. The first step is consistent with the release of the water molecule (calc. 3.00%, found at 2.55%). Two chlorine atoms were eliminated in the second stage (calculated at. 11.83% found at 11.47%). According to the mass loss analysis, the third stage is a dissociation of the AMPY (calc. 18.21%, found 17.98%). At 343°C, the DTA trace has an endothermic action. The remaining resulting deterioration was assigned based on mass loss consideration to the formation of 1/2 V_2_O_3_ (calc.11.17%, found at 10.92%). The DTA curve has an exothermic peak at 411°C (Figure 7(a)).

#### 3.4.2. [Fe(HL)(AMPY)(H_2_O)Cl_2_]

The measured mass loss of this early stage and the loss of one water molecule (calc. 2.98%, found at 2.52%) are highly connected. The observed mass drop in the second step is due to two chlorine atoms (calc. 11.73%, found at 11.40%). An endothermic DTA peak at 256°C corresponds to this stage. The breakdown of the remaining ligands is shown in the final step (calc. 76.07%, discovered 74.92%). On the DTA curve, this stage is shown by a significant exothermic peak at 358°C. 1/2 Fe_2_O_3_ lasted for a stable residue and was found to be 11.57%, compared to 11.89% calculated (Figure 7(b)).

#### 3.4.3. [Ni(HL)(AMPY)(H_2_O)Cl_2_]

The TGA and DTA curves of the sample Ni(II) complex are shown in Figure 7(c). The thermal degradation processes, which have *four* steps, start at a heat of 76°C, as evidenced by the TG-analysis findings. The first mass loss is closely related to the discharge of water molecules. The mass loss (calc. 2.96%, found at 2.75%) points to the loss of the H_2_O molecule. The DTA curve has an endothermic peak at 80°C. Two chlorine atoms were removed during the second breakdown stage (142–248°C; calculated 11.68%, found 11.31%). The third step discarded the AMPY ligand, and the fourth stage represented the decomposition of the rest complex, respectively (calculated at 17.98% and found 17.64%), and (calculated at 57.74% and found at 57.53%) on the DTA curve, and there are endothermic peaks at 318°C and exothermic peaks at 450°C. At 550°C, the finished item is consistent with NiO (calculated at 12.30%, found at 11.72%).

### 3.5. X-Ray Diffraction (XRD)

**Figure 8:**
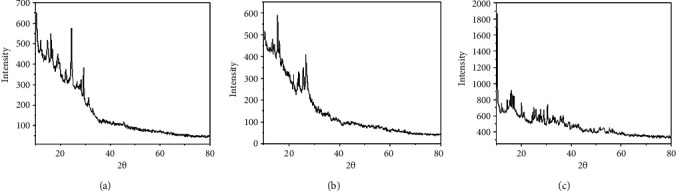
XRD pattern of (a) V(III), (b) Fe(III), and (c) Ni(II) compounds, respectively.

For powder X-ray diffraction experiments, the vanadium(III), iron(III), and nickel (II) complexes were used. The complex diffraction patterns are captured between 2*θ*, spanning 10° to 80°. Scherrer's formula is used to calculate the samples' particle sizes. Scherrer's equation states that the particle size is provided by the formula *t* = 0.9 *λ*/Bcos*θ*, where the crystal's thickness (in nm) is *t*, *B* stands for half width (measured in radians), *θ* for the Bragg angle, and *λ* for the wavelength. The measurement of the diffraction peak's half-width yields the particle size corresponding to each diffraction maxima.  Table 5 displays the value of the lattice parameter as well as the particle size for each of the three complexes. The V(III) and Fe(III) complexes are triclinic, while that of the Ni(II) complex is a system of monoclinic crystals. Sharp peaks can be seen in all metal complexes. The particle sizes of all complexes were found to be between 21 and 49 nm (Figure 8).

### 3.6. SEM Morphological Study

**Figure 9:**
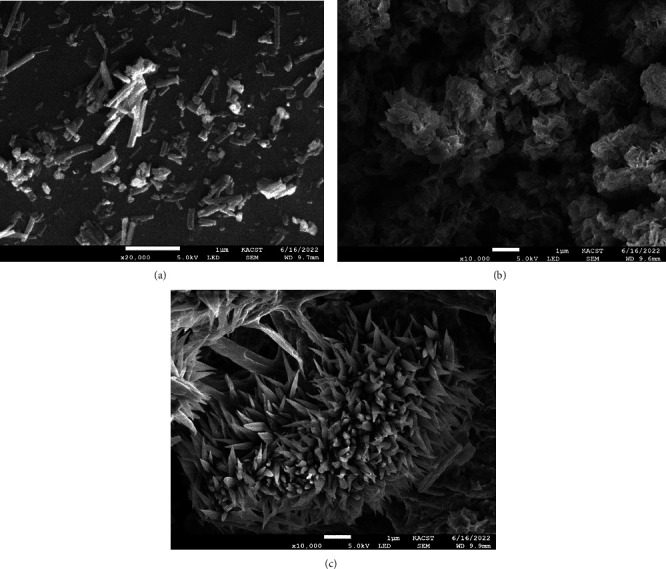
SEM micrographs of (a) V(III), (b) Fe(III), and (c) Ni(II) complexes.

The microstructure and surface appearance of the V(III), Fe(III), and Ni(II) complexes were studied using scanning electron microscopy. The shape of the particles of the V(III) complex particles' shape is consistent with the nanorod structure (Figure 9(a)). The morphologies of cauliflower and coral, respectively, are seen for the substances Fe(III) and Ni(II) (Figures 9(b) and 9(c)).

### 3.7. Antimicrobial Activity Study

Table 6 provides the in vitro antimicrobial screening results. Bacterial strains, including (*Bacillus subtilis*) which are Gram-positive and two Gram-negative strains (*E-coli and Pseudomonas aeruginosa*), were used to test the antimicrobial actions (HL), (AMPY) of the free ligand and complexes of vanadium(III), iron(III), and nickel(II). They were also tested against the fungus (*Aspergillus fumigatus*).

#### 3.7.1. Antibacterial and Antifungal Screening

**Figure 10:**
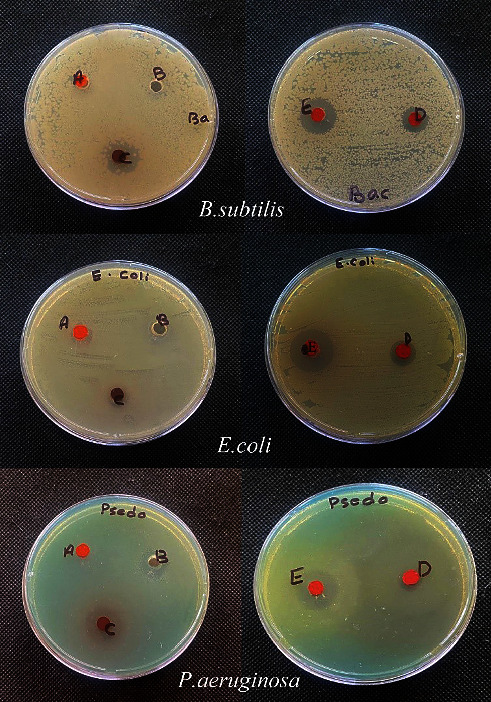
Antibacterial activity of complexes (A, B, C, D, E) against values of *B. subtilis*, *E. coli*, and *P. aeruginosa* inhibition zones (mm).

**Figure 11:**
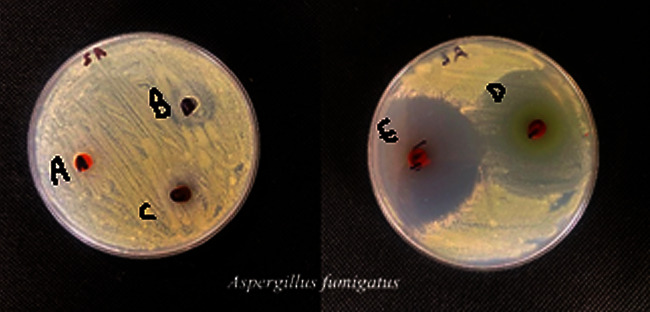
Antifungal activity of complexes (A, B, C, D, E) against *A. fumigatus* values of inhibition zone (mm).

**Figure 12:**
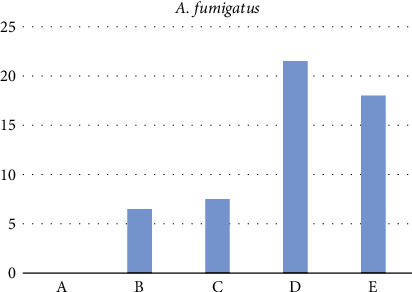
A representation of HL ability to fight AMPY ligands of the fungus in addition to three metal compounds in a histogram (^A^HL ligand, ^B^AMPY ligand, ^C^Fe(III) complex, ^D^V(III) complex, and ^E^Ni(II) complex).

According to the findings, all complexes are effective in opposition to all examined species. These numbers exceed those for free HL and AMPY ligands, where the metal complexes showed better activity compared to the original ligands [19]. The most active ligand complexes were those containing nickel and vanadium, while the least active ligand complexes contained iron (Figure 10). It is clear from the antibacterial data that coordination with the metal atoms increased the activity of the Schiff base and AMPY. The results of the susceptibility of these bacteria to chemicals determined by the diameter of the inhibition zone (IZ) are shown in Table 6 for fungi. A clear area surrounding the disc indicates that the substance has an inhibitory effect on an organism, in the order as follows: vanadium (III) > nickel (II) > iron (III) (Figures 11 and 12). Metal complexes were more energetic than ligands; thus, chelation theory might be able to explain this rise in activity. This was as a result of chelation's ability to decrease the polarity of the metal ion by sharing some of its positive charge with the donor groups [30].

## 4. Conclusions

**Figure 13:**
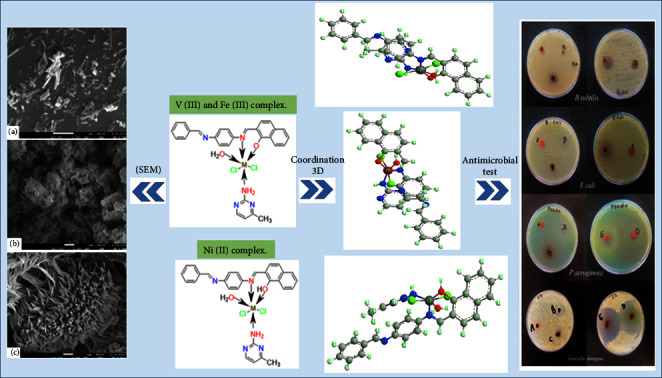
A perspective view of complete coordination, SEM micrographs, and their antimicrobial activity.

**Figure 14:**
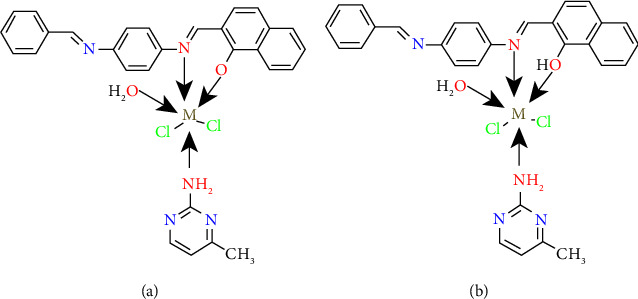
M: (a) Fe(III) and V(III). (b) Ni(II).

In the present study, three transition metal (II) or (III) complexes were synthesized and characterized by various spectral and physicochemical techniques. The coordination of the first ligand (Schiff base) occurred through the phenolic -OH group and a nitrogen atom of an azomethine group (-C=N-), while the coordination of the second ligand (AMPY) occurred through the NH_2_ group, according to the spectrum data. According to the suggestions acquired from overhead readings (elemental analysis, magnetic susceptibility, molar conductance, UV-visible, FTIR, TGA analysis, and XRD tests), we propose an octahedral structure for V(III), Fe(III), and Ni(II) complexes with HL ligand and other AMPY ligand. The V(III), Fe(III), and Ni(II) complexes, respectively, are shown in Figures 13 and 14 below, while the Fe(III) complex's particles have a cauliflower shape, and those of the V(III) complex have a nanorod shape. The Ni(II) compound causes coral-like morphologies, and the molar conductivity of the complexes has the nonelectrolytic shape of all the complexes. In vitro, the effectiveness of the free ligands and their metal complexes against pathogenic organisms was tested, and the results showed that the complexes had more activity compared to the free ligands.

Based on the results in the current manuscript, the substance has an inhibitory effect on an organism, in the order as follows: vanadium (III) > nickel (II) > iron (III).

## Figures and Tables

**Table 1:** The complexes' color, molar conductance, elemental analysis, and melting points.

Compound	Formula (M.Wt)	Color	M.P.(°C) Dec	Yield (%)	Elemental analyses found (calculated)%			*∧* ^*∗*^
					*C*%	*H*%	*N*%	
L1	C_24_H_18_N_2_O (350.41)	Orange	230	84	—	—	—	—
L2	C_5_H_7_N_3_ (109.13)	Colorless	158		—	—	—	—
V(III) complex	C_29_H_26_N_5_O_2_VCl_2_ (598.11)	Red	260	70	58.42 (58.23)	4.74 (4.39)	11.96 (11.71)	20.6
Fe(III) complex	C_29_H_26_N_5_O_2_FeCl_2_ (603.01)	Black	>300	82	57.52 (57.75)	4.96 (4.35)	11.79 (11.61)	30.5
Ni(II) complex	C_29_H_27_N_5_O_2_NiCl_2_ (606.86)	Brick red	>300	75	57.84 (57.39)	4.74 (4.49)	11.62 (11.54)	32.2

dec. = decomposition. *∧*^*∗*^ = molar conductivity, Λm Scm^2^ mol^−1^.

**Table 2:** FT-IR spectral data (cm^−1^) of free ligands and their metal complexes.

Comp.	*υ* (OH) coordinated water	*υ* (NH_2_)	*δ* (NH_2_)	*δ* (OH) in-plane	*υ* (C=N)	*υ* (C=C)	*υ* (C-O)	*υ* (M-O)	*υ* (M-N)	*υ* (M-cl)
L1 (HL)	—	—	—	850	1618-1604	1540	1310	—	—	—
L2 (AMPY)	—	3430-3305	1648	—	—	—	—	—	—	—
V(III) complex	3330	Disappear	1665	832	1613-1545	1514	1303	623	532	430
Fe(III) complex	3330	Disappear	1656	826	1614-1594	1513	1305	640	544	442
Ni(II) complex	3423	3415-3286	1661	823	1600-1560	1546	1304	627	566	429

**Table 3:** Absorption spectra of L1 and L2 ligands with their compounds and magnetic moments.

Compound	*λ* max. (nm)	*λ* max. (cm^−1^)	Assignment	Meff (B.M)	*d* ^*n*^	Geometry
L1 (HL)	259	38,610	*π* ⟶ *π*^*∗*^	—	—	—

L2 (AMPY)	280	35,714	*n* ⟶ *π*^*∗*^	—	—	—

V(III) *complex*	488	20,492	^3^T_1_ (F) ⟶ ^3^T_1_ (P)	2.88	*d* ^2^	*O*_*h*_
	320	31,250	*n* ⟶ *π*^*∗*^			
	266	37,594	*π* ⟶ *π*^*∗*^			

Fe(III) *complex*	489	20,450	^4^T_1g_ (D) ⟶ ^6^A_1g_	5.96	*d* ^5^	*O*_*h*_
	463	21,598	^4^T_2g_ (G) ⟶ ^6^A_1g_			
	390	25,641	^4^T_2g_ (G) ⟶ ^6^A_1g_			
	300	33,333	*n* ⟶ *π*^*∗*^			
	267	37,453	*π* ⟶*π*^*∗*^			

Ni(II) *complex*	470	21,277	^3^A_2g_ (F) ⟶ ^3^T_1g_ (P)	2.92	*d* ^8^	*O*_*h*_
	303	33,003	*n* ⟶ *π*^*∗*^			
	260	38,462	*π* ⟶ *π*^*∗*^			

BM = Bohr magneton. *O*_*h*_ _=_ octahedral.

**Table 4:** Thermal decomposition data of metal compounds.

Compound	Step	Temp. range °C	Assignment	TGA (Wt. loss %) Found (calcd.)
V(III) complex	1^st^	82–155	Loss of coordinated water molecules (H_2_O)	2.55 (3.00)
	2^nd^	157–260	Loss of two chloride atoms	11.47 (11.83)
				17.98 (18.21)
	3^rd^	262–348	Loss of ligand (AMPY)	58.12 (58.48)
	4^th^	350–550	Decomposition with the formation of final product vanadium oxide	10.92 (11.17)

Fe(III) complex	1^st^	80–162	Loss of coordinated water molecules (H_2_O)	2.52 (2.98)
	2^nd^	164–260	Loss of two chloride atoms	11.40 (11.73)
	3^rd^	262–550	Thermal decomposition of the rest of the complex and forming iron oxide	74.92 (76.07)
				11.57 (11.89)

Ni(II) complex	1^st^	76–140	Loss of coordinated water molecules (H_2_O)	2.75 (2.96)
				11.31 (11.68)
	2^nd^	142–248	Loss of two chloride atoms	17.64 (17.98)
	3^rd^	250–347	Loss of ligand (AMPY)	57.53 (57.74)
	4^th^	349–550	Thermal decomposition of the rest of the complex, and forming nickel oxide	11.72 (12.30)

**Table 5:** The crystal data of the complexes.

Parameters	V(III) complex	Fe(III) complex	Ni(II) complex
Empirical formula	C_29_H_27_N_5_O_2_VCl_2_	C_29_H_27_N_5_O_2_FeCl_2_	C_29_H_27_N_5_O_2_NiCl_2_
Formula weight	599.11	604.01	606.86
Crystal system	Triclinic	Triclinic	Monoclinic
*a* (Å)	3.822	4.630	6.704
*b* (Å)	7.389	7.578	11.843
*c* (Å)	10.138	8.852	12.63
Alfa (°)	71.593	79.080	90.00
Beta (°)	52.656	113.732	101.2
Gamma (°)	53.170	46.041	90.00
Volume of unit cell (Å3)	181.66	202.77	983
Particle size (nm)	29	50	39

**Table 6:** The antimicrobial activity of the complexes (A, B, C, D, E) against inhibition zone values of *B. subtilis*, *E. coli*, *P. aeruginosa*, and *A. fumigatus*.

	Halo of inhibition mm			
	*Bacillus subtilis*	*Escherichia coli*	*Pseudomonas aeruginosa*	Aspergillus fumigatus (fungus)
A	0	2	0	0
B	0	2.5	3.5	6.5
C	6.5	3.5	5	7.5
D	7	4	8.5	21.5
E	8	9	7.5	18

^A^HL ligand, ^B^AMPY ligand, ^C^Fe(III) complex, ^D^V(III) complex, and ^E^Ni(II) complex.

## Data Availability

The data used to support the findings of this study are available from the corresponding author upon request.

## Supplementary Materials

Supplementary Materials SCHEME S1: decomposition steps of the compound V(III), SCHEME S2: decomposition steps of the compound Fe(III), and SCHEME S3: decomposition steps of Ni(II) compound. Figure S1: FT-IR spectrum of the ligand (HL). Figure S2: FT-IR spectrum of the ligand (AMPY). Figure S3: FT-IR spectra of V(III) complex. Figure S4: FT-IR spectra of Fe(III) complex. Figure S5: FT-IR spectra of Ni(II) complex.

## References

[1] Al-Fakeh M. S., Messaoudi S., Alresheedi F. I., Albadri A. E. A. E., El-Sayed W. A., Saleh E. E. (2023). Preparation, characterization, DFT calculations, antibacterial and molecular docking study of Co(II), Cu(II), and Zn(II) mixed ligand complexes. *Crystals*.

[2] Zayed E. M., Mohamed G. G., Abd El Salam H. A. (2023). Ni(II), Co(II), Fe(III), and Zn(II) mixed ligand complexes of indoline-dione and naphthalene-dione: synthesis, characterization, thermal, antimicrobial, and molecular modeling studies. *Inorganic Chemistry Communications*.

[3] Al-Fakeh M. S., Alsikhan M. A., Alnawmasi J. S. (2023). Physico-chemical study of Mn(II), Co(II), Cu(II), Cr(III), and Pd(II) complexes with schiff-base and aminopyrimidyl derivatives and anti-cancer, antioxidant, antimicrobial applications. *Molecules*.

[4] Deswal Y. (2024). Metal complexes of 1, 2, 4-triazole based ligand: synthesis, structural elucidation, DFT calculations, alpha-amylase and alpha-glucosidase inhibitory activity along with molecular docking studies. *Journal of Inorganic and Organometallic Polymers and Materials*.

[5] Matela G. (2020). Schiff bases and complexes: a review on anti-cancer activity. *Anti-Cancer Agents in Medicinal Chemistry*.

[6] El-Saied F. A., Salem T. A., Shakdofa M. M. E., Al-Hakimi A. N., Radwan A. S. (2018). Antitumor activity of synthesized and characterized Cu(II), Ni(II) and Co(II) complexes of hydrazone-oxime ligands derived from 3-(hydroxyimino) butan-2-one. *Beni-Suef University Journal of Basic and Applied Sciences*.

[7] Deswal Y., Asija S., Tufail A. (2023). Instigating the in vitro antidiabetic activity of new tridentate Schiff base ligand appended M (II) complexes: from synthesis, structural characterization, quantum computational calculations to molecular docking, and molecular dynamics simulation studies. *Applied Organometallic Chemistry*.

[8] Agarwal P., Asija S., Deswal Y., Kumar N. (2022). Recent advancements in the anticancer potentials of first row transition metal complexes. *Journal of the Indian Chemical Society*.

[9] Al Zoubi W., Al-Hamdani A. A. S., Ahmed S. D., Ko Y. G. (2018). Synthesis, characterization, and biological activity of Schiff bases metal complexes. *Journal of Physical Organic Chemistry*.

[10] Chaudhry A. H. (2013). A review (part a) – general applications of Schiff base transition metal complexes. *International Journal of Current Pharmaceutical Research*.

[11] Bayrak C. (2013). Vibrational spectroscopic investigation of 2-amino-4-methylpyrimidine tetracyanonickelate complexes. *Hacettepe J. Biol. Chem.*.

[12] Revathi N., Sankarganesh M., Dhaveethu Raja J., Vinoth Kumar G. G., Sakthivel A., Rajasekaran R. (2021). Bio-active mixed ligand Cu(II) and Zn(II) complexes of pyrimidine derivative Schiff base: DFT calculation, antimicrobial, antioxidant, DNA binding, anticancer and molecular docking studies. *Journal of Biomolecular Structure and Dynamics*.

[13] Jaziri E., Louis H., Gharbi C. (2022). Synthesis, X-ray crystallography, molecular electronic property investigation, and leukopoiesis activity of novel 4, 6-dimethyl-1, 6-dihydropyridin-2-amino nitrate hybrid material. *Journal of Molecular Structure*.

[14] Demehin A. I., Oladipo M. A., Semire B. (2020). Synthesis, spectroscopic, biological activities and DFT calculations of nickel(II) mixed-ligand complexes of tridentate Schiff bases. *Eclética Química Journal*.

[15] Vairalakshmi M., Princess R., Rani B. K., Raja S. J. (2018). Synthesis, structural elucidation, catalytic, antibacterial and antioxidant activity of thiophene derived mixed ligand metal complexes. *Journal of the Chilean Chemical Society*.

[16] Aly A. A., Al-Fakeh M. S., Ghandour M. A., Abu-Zied B. M. (2013). New nano-sized supramolecular metal coordination polymers derived from 1, 2-bis (2-pyridyl)-ethene and benzimidazole. *Nano Sci. Nano Technol. Indian J. NSNTAIJ*.

[17] Wang J. X., Roush M. L. (2020). \ (3) 1(2), what every eng. Should know about risk. *Engineering Management*.

[18] Vidhu V. K., Aromal S. A., Philip D. (2011). Green synthesis of silver nanoparticles using Macrotyloma uniflorum. *Spectrochimica Acta Part A: Molecular and Biomolecular Spectroscopy*.

[19] Dos Santos A. P., Da Fonseca M. G., De Paiva Espínola J. G., De Oliveira S. F., Arakaki L. N. H. (2005). Adducts of antimony triiodide and 2-aminomethylpyridines: synthesis, characterization and thermochemistry. *Thermochimica Acta*.

[20] Nassirinia N., Shahmoradi G., Sadeghy N., Amani S. (2007). Synthesis, spectroscopic and magnetic properties of three dinuclear copper(II) complexes [Cu_2_(2-amino-4-methylpyrimidine)_4_(OH)_2_](X)_2_, where X **=** , or. *Journal of Coordination Chemistry*.

[21] Hasanein A. A., El-Subruiti G. M., Younes G. O., Srour M. H. (2004). Spectral studies on some pyrimidine derivatives in different solvents. *Journal of Solution Chemistry*.

[22] Altameemi A., Abid K., Al-Bayati S., Rasheed A. (2016). Synthesis, characterization, thermal study and biological evaluation of transition metal complexes supported by ONNNO-pentadentate Schiff base ligand qumarine derivative view project acyclic Schiff base complexes view project synthesis, characterization. *American Journal of Chemistry*.

[23] Baboo M., Ahmed S., College H. (2013). A comprehensive analysis of vanadium (III) and its complexes with Schiff bases. *IJRES*.

[24] Mahmoud W. H., Omar M. M., Ahmed Y. M., Mohamed G. G. (2020). Transition metal complexes of Schiff base ligand based on 4,6-diacetyl resorcinol. *Applied Organometallic Chemistry*.

[25] El-Ghoul Y., Al-Fakeh M. S., Al-Subaie N. S. (2023). Synthesis and characterization of a new alginate/carrageenan crosslinked biopolymer and study of the antibacterial, antioxidant, and anticancer performance of its Mn (II), Fe (III), Ni (II), and Cu (II) polymeric complexes. *Polymers*.

[26] Kudrat-E-Zahan M., Islam M. S., Abul Bashar M. (2015). Synthesis, characteristics, and antimicrobial activity of some complexes of Mn(II), Fe(III) Co(II), Ni(II), Cu(II), and Sb(III) containing bidentate Schiff base of SMDTC. *Russian Journal of General Chemistry*.

[27] Golovnev N. N., Molokeev M. S., Sterkhova I. V., Lesnikov M. K. (2018). Two novel mixed-ligand Ni(II) and Co(II) complexes with 1,10-phenanthroline: synthesis, structural characterization, and thermal stability. *Chemical Physics Letters*.

[28] Jahan R. (2017). Preparation and characterization of Ni (II) transition metal mixed ligand complexes. *Transition*.

[29] Adekunle Ajayeoba T. (2019). Synthesis, characterisation and acetylcholinesterase inhibition activity of nickel(II) and copper(II) complexes of 3-Hydroxybenzaldehyde-4-nitrobenzoic acid hydrazone. *American Journal of Applied Chemistry*.

[30] El-Sonbati A. Z., Mahmoud W. H., Mohamed G. G., Diab M. A., Morgan S. M., Abbas S. Y. (2019). Synthesis, characterization of Schiff base metal complexes and their biological investigation. *Applied Organometallic Chemistry*.

