# Corrigendum to “New Nanosized V(III), Fe(III), and Ni(II) Complexes Comprising Schiff Base and 2-Amino-4-Methyl Pyrimidine: Synthesis, Properties, and Biological Activity”

**DOI:** 10.1155/2024/9896516

**Published:** 2024-09-18

**Authors:** Maged S. Al-Fakeh, Maha A. Alsikhan, Jawza Sh Alnawmasi, Mona S. Al-Wahibi

**Affiliations:** ^1^Department of Chemistry, College of Science, Qassim University, Buraydah 51452, Saudi Arabia; ^2^Taiz University, Taiz 3086, Yemen; ^3^Department of Botany and Microbiology, College of Science, King Saud University, Riyadh 2455, Saudi Arabia

In the article titled “New Nanosized V(III), Fe(III), and Ni(II) Complexes Comprising Schiff Base and 2-Amino-4-Methyl Pyrimidine: Synthesis, Properties, and Biological Activity” [1], Abdullah H. Alluhayb was mistakenly included as an author without his consent. Abdullah H. Alluhayb did not contribute to the research being reported or the preparation of the article. Based on his request, his name has been removed from the author list. The corrected author list can be found above.
