# Supplementary material for: New Nanosized V(III), Fe(III), and Ni(II) Complexes Comprising Schiff Base and 2-Amino-4-Methyl Pyrimidine: Synthesis, Properties, and Biological Activity
Source: Int J Biomater. 2024 May 14;2024:9198129. doi: 10.1155/2024/9198129 (PMC11390230; doi:10.1155/2024/9198129)
Supplement: Supplementary Materials — SCHEME S1: decomposition steps of the compound V(III), SCHEME S2: decomposition steps of the compound Fe(III), and SCHEME S3: decomposition steps of Ni(II) compound. Figure S1: FT-IR spectrum of the ligand (HL). Figure S2: FT-IR spectrum of the ligand (AMPY). Figure S3: FT-IR spectra of V(III) complex. Figure S4: FT-IR spectra of Fe(III) complex. Figure S5: FT-IR spectra of Ni(II) complex. [file 9198129.f1.docx]

**Supplementary File**

SCHEME S1: Decomposition steps of the compound **V(III)**, SCHEME S2: Decomposition‎ steps of the compound **Fe(III)**, and SCHEME S3: Decomposition‎ steps of **Ni(II)** compound. Figure S1: ^1^H NMR spectrum of ligand HL.

Figure S2: ^13^C NMR spectrum of ligand HL. Figure S3: FT-IR spectrum of the ligand (HL). Figure S4: FT-IR spectrum of the ligand (AMPY). Figure S5: FT-IR spectra of V(III) complex. Figure S6: FT-IR spectra of Fe(III) complex. Figure S7: FT-IR spectra of Ni(II) complex.

SCHEME S1: Decomposition steps of the compound **V(III)**.

SCHEME S2: Decomposition‎ steps of the compound **Fe(III)**.

SCHEME S3: Decomposition‎ steps of **Ni(II)** compound.


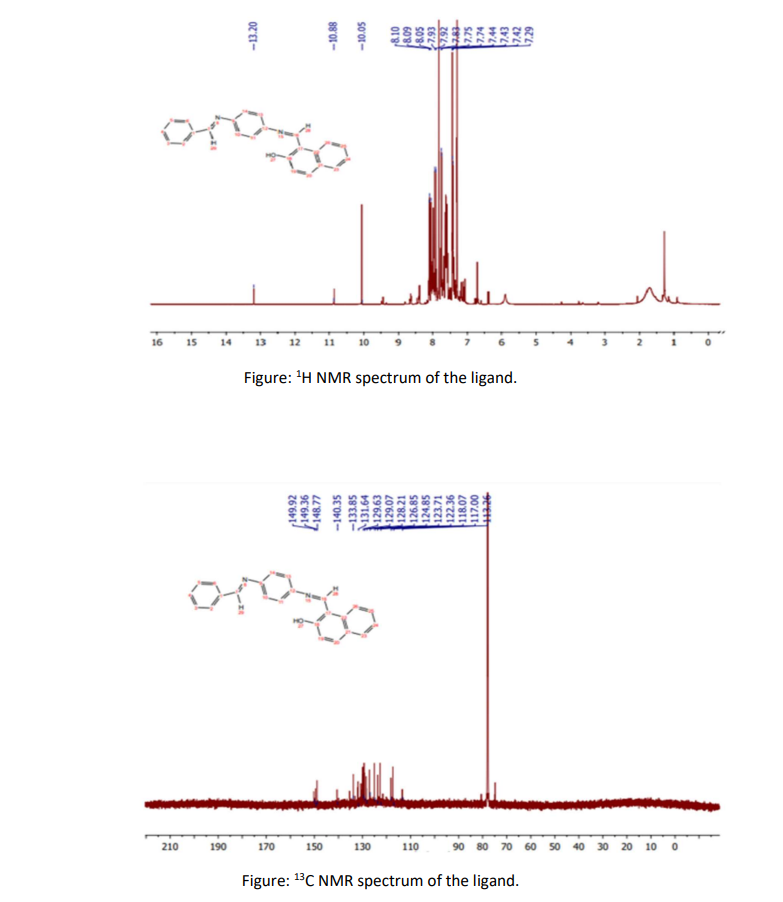


Figure S1: ^1^H NMR spectrum of ligand HL.


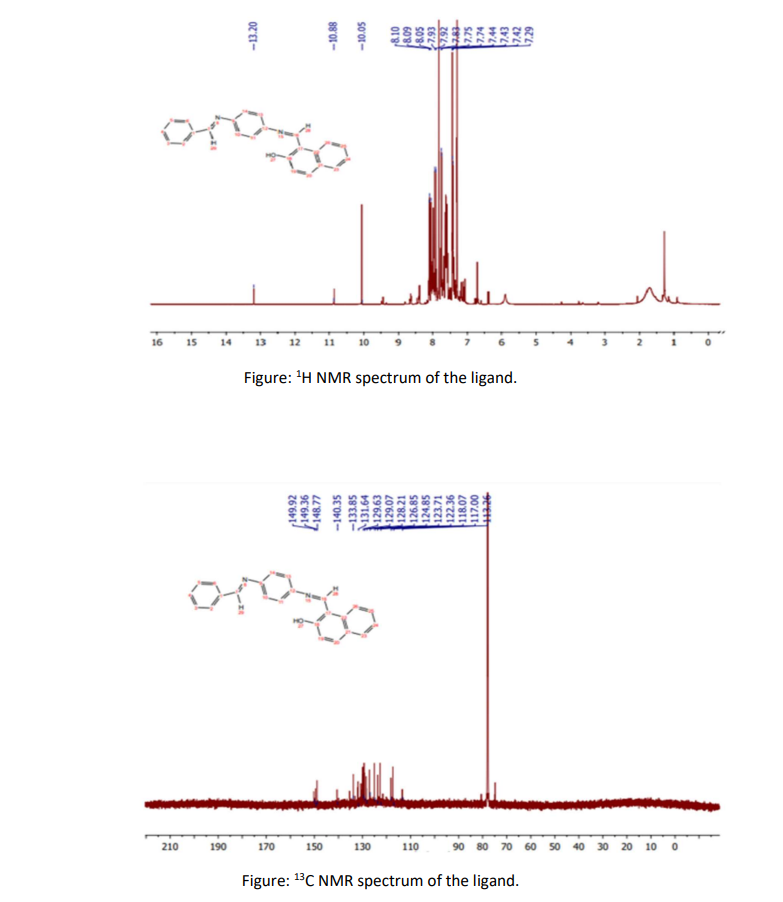


Figure S2: ^13^C NMR spectrum of ligand HL.


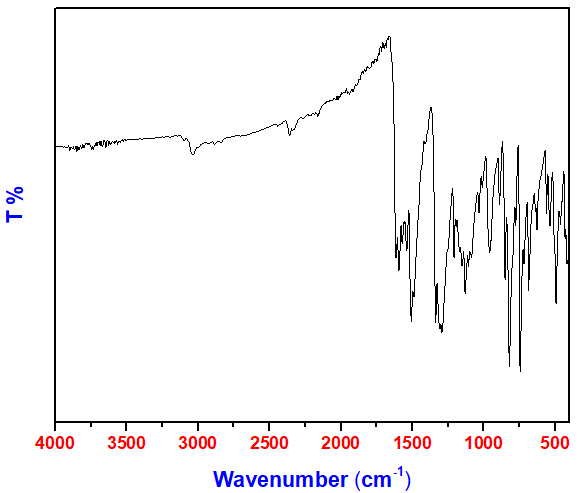


Figure S3: FT-IR spectrum of the ligand (HL).‎


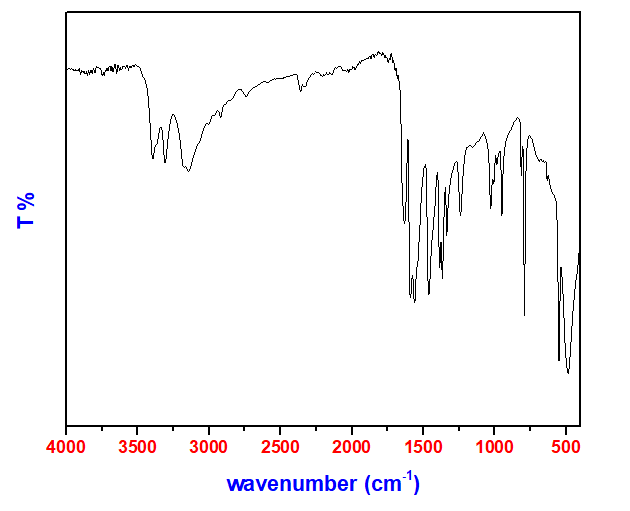


Figure S4: FT-IR spectrum of the ligand (AMPY).

‎

‎


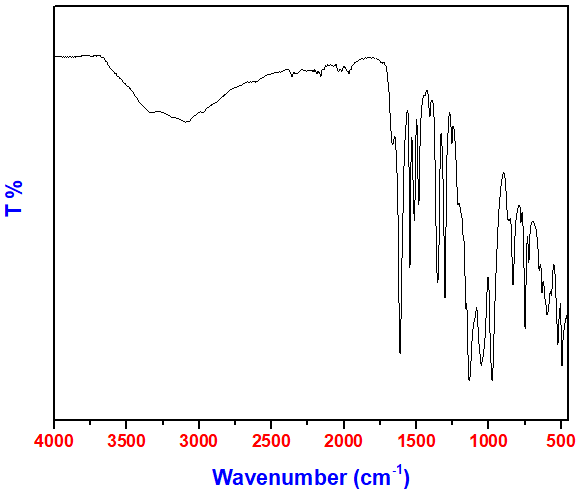


Figure S5: FT-IR spectra of V(III) complex.‎


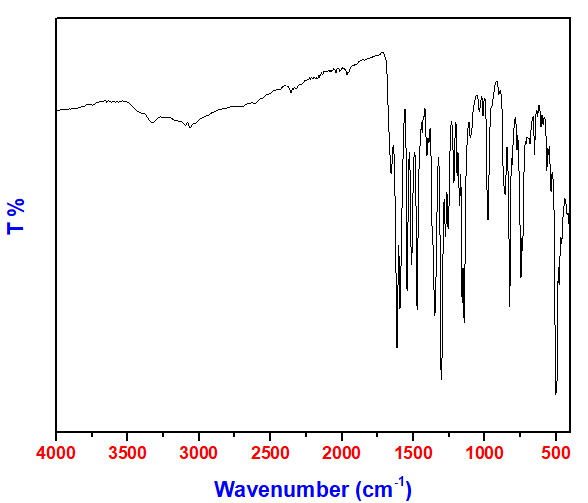


Figure S6: FT-IR spectra of Fe(III) complex.‎


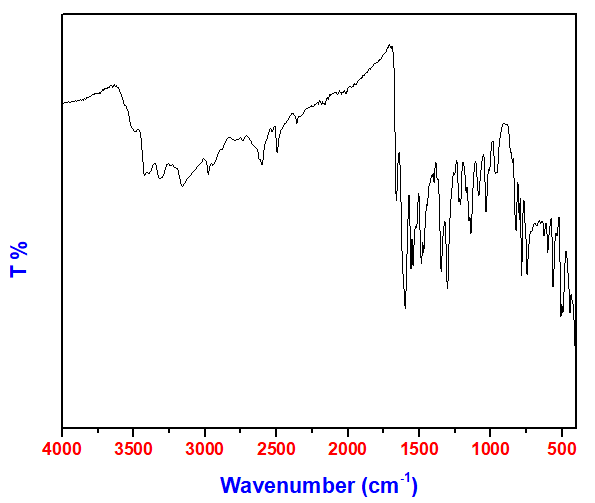


Figure S7: FT-IR spectra of Ni(II) complex.
